# Application of microalgae *Scenedesmus acuminatus* enhances water quality in rice-crayfish culture

**DOI:** 10.3389/fbioe.2023.1143622

**Published:** 2023-05-04

**Authors:** Danni Yuan, Lan Wang, Hongxia Wang, Rongli Miao, Yulu Wang, Hu Jin, Lu Tan, Chaojun Wei, Qiang Hu, Yingchun Gong

**Affiliations:** ^1^ School of Environmental Ecology and Biological Engineering, Wuhan Institute of Technology, Wuhan, China; ^2^ Center for Microalgal Biotechnology and Biofuels, Institute of Hydrobiology, Chinese Academy of Sciences, Wuhan, China; ^3^ State Key Laboratory of Freshwater Ecology and Biotechnology, Institute of Hydrobiology, Chinese Academy of Sciences, Wuhan, China; ^4^ Hydrobiological Data Analysis Center, Institute of Hydrobiology, Chinese Academy of Sciences, Wuhan, China; ^5^ Systems Ecology and Watershed Ecology Center for Freshwater Ecology, Institute of Hydrobiology, Chinese Academy of Sciences, Wuhan, China; ^6^ CAS Key Laboratory of Quantitative Engineering Biology, Shenzhen Institute of Synthetic Biology, Shenzhen Institute of Advanced Technology, Chinese Academy of Sciences, Shenzhen, China; ^7^ Faculty of Synthetic Biology, Shenzhen Institute of Advanced Technology, Chinese Academy of Sciences, Shenzhen, China

**Keywords:** microalgal application, aquaculture ecosystem, water quality, *Spirogyra*, sustainability

## Abstract

Improper management of aquatic environments substantially restricts the development of the aquaculture industry. The industrialisation of the crayfish *Procambarus clarkii*, for example, is currently being limited by poor water quality. Research suggests that microalgal biotechnology has a great potential for water quality regulation. However, the ecological effects of microalgal applications on aquatic communities in aquaculture systems remain largely unknown. In the present study, 5 L *Scenedesmus acuminatus* GT-2 culture (biomass 120 g L^-1^) was added to an approximately 1,000 m^2^ rice-crayfish culture to examine the response of aquatic ecosystems to microalgal application. The total nitrogen content decreased significantly as a result of microalgal addition. Moreover, the microalgal addition changed the bacterial community structure directionally and produced more nitrate reducing and aerobic bacteria. The effect of microalgal addition on plankton community structure was not obvious, except for a significant difference in *Spirogyra* growth which was inhibited by 81.0% under microalgal addition. Furthermore, the network of microorganisms in culture systems with the added microalga had higher interconnectivity and was more complex, which indicating microalgal application enhance the stability of aquaculture systems. The application of microalgae was found to have the greatest effect on the 6th day of the experiment, as supported by both environmental and biological evidence. These findings can provide valuable guidance for the practical application of microalgae in aquaculture systems.

## 1 Introduction

The decapod *Procambarus clarkii*, commonly known as crayfish or red swamp crayfish, is a popular commercially reared aquatic species in China because of its delicious meat and rich nutritional value. In 2021, 2.63 million t of *P. clarkii* crayfish were cultivated, accounting for 50%–60% of the total cultivated freshwater crustaceans (Yearbook, 2022), with the rice–crayfish model being the dominant model for farming. However, the continuous expansion of artificial breeding areas and the inadequacy of water quality control technologies have resulted in concerning problems, such as mass growth of filamentous algae (mainly *Spirogyra* species), white spot syndrome virus, and pathogenic bacteria (such as *Aeromonas*) (Gao et al., 2020; Liu et al., 2021). Traditional prevention and treatment methods for these issues mainly rely on chemical drugs and antibiotics, which have several limitations and drawbacks, such as drug residues, environmental pollution, immune suppression, and destruction of micro-ecological balance. In addition, the main method of *Spirogyra* removal is manual salvage, which is not only time-consuming and laborious but may also be ineffective, since it is difficult to remove *Spirogyra* completely, so repeated outbreaks of the alga may occur. Thus, there is a need for safe, environmentally friendly, and effective measures to maintain the ecological balance of pond ecosystems for sustainable aquaculture production.

Owing to its economic and ecological benefits, the application of microalgal biotechnology in aquaculture systems has recently attracted attention (Li, et al., 2019; Lu, et al., 2020). Microalgae exhibit great potential for nutrient removal and bioremediation. Zhang et al. (2019) reported that *Spirulina* can efficiently use nutrients, as well as control the water quality and reduce the water usage in high-density fish farms where water exchange is limited. Kumar et al. (2016) reported that the marine microalga *Picochlorum maculatum* can remove 89.6% nitrite, 98.5% ammonia, 57.0% phosphate, and 46.4% nitrate from a shrimp culture system.

However, although water quality and product output variations have been the main focus to date, the ecological structure of pond systems during the process of microalgal application in aquaculture remains unexplored. The biological elements should be considered as organisms and the environment are interconnected and often interact with each other (Naiman et al., 1999; Garnier et al., 2020). These interactions in aquatic communities are significant and should not be overlooked. A comprehensive consideration of the ecological factors in the aquaculture system is necessary to understand the mechanisms of microalgal application technology and to promote and optimize the technology. To achieve successful ecological regulation of aquaculture systems, a stable ecosystem that can self-purify should be cultivated and maintained (Ramli et al., 2018). Consequently, it is crucial to find a way to stabilise aquaculture ecosystems for the sustainable development of aquaculture.

Additionally, previous studies on the application of microalgal technology have focused on small systems in the laboratory, and the effect of this technology on outdoor aquaculture systems remains unknown. Based on the above evidence, this study hypothesised that microalgal application would influence the water quality, microorganism community and ecosystem stability in the crayfish *P*. *clarkii* cultures. Therefore, we designed an experiment in which high density *Scenedesmus acuminatus* solution was applied to a rice-crayfish culture. *S. acuminatus* GT-2 was selected for this experiment, as it is cosmopolitan and one of the most common genera of green microalgae in freshwater environments. Additionally, it is also one of the foods consumed by omnivorous crayfish, including *P. clarkii* (Alcorlo et al., 2004; unpublished data). Moreover, it can be effective at removing nitrogen and phosphorus from aqueous media under different pollution conditions (Tejido-Nunez et al., 2019; Oliveira et al., 2021). The effect of microalgal application on water quality, species composition and stability of the aquaculture systems was evaluated. This study will promote the application of microalgae in aquaculture systems and accelerate the development of sustainable aquaculture.

## 2 Materials and methods

### 2.1 Microalgal cultivation

To quickly obtain a high-density and pollution-free microalgal solution, fermentation was selected as the cultivation method. Two 50-L fermenters (Biotech-50BS, China) were used for heterotrophic cultivation of *S. acuminatus* GT-2 according to the cultivation method described by Jin et al. (2020). The initial working volume of the cultivation was 10 L. During the fermentation process, a 1 M HCl or 3 M NaOH solution was used to maintain the pH, and aeration was maintained at 1 vvm with an airflow rate of 12 L min^-1^. Dissolved oxygen (DO) was controlled automatically to remain above 40% by adjusting the stirring speed. In the fermentor batch medium, KNO_3_ was replaced by 0.84 g L^-1^ of urea. The glucose concentration in the fermenter was carefully controlled to optimize the growth of *S. acuminatus* GT-2 within the range of 0–5 g L^-1^. After 5 days of fermentation in the laboratory, the harvested alga was immediately transported to the experimental base.

### 2.2 Experimental design in the field

The experiment was conducted at the experimental facilities of Huazhong Agriculture University, Jingzhou, China (39°67′N, 116°58′E) between 1 and 26 May 2019. Field banks divided a rice-crayfish culture (approximately 4,000 m^2^) into four ponds, with a water depth of approximately 0.50 m and a volume of approximately 5×10^5^ L. Two of these ponds were selected haphazardly to maintain the existing state of the crayfish culture without microalgae added (no-MA group). The other two ponds were selected to add 5 L of *S. acuminatus* GT-2 culture (biomass 120 g L^-1^) to the existing crayfish culture (MA group). Two sampling points were set in each pond. The working concentration of *S*. *acuminatus* was approximately 1.20 mg L^-1^ in each microalgae added pond. The stocking density for crayfish is approximately 20 individuals per square meter, with an average weight of 15 g per crayfish. The crayfish were fed twice a day, in the morning and evening, with a specialized crayfish feed containing 32% protein, produced by Haida Group Co., Ltd. The feeding amount was adjusted based on the feeding behavior of the crayfish, and the feeding rate was kept consistent across all experimental ponds. Water samples were collected from the two groups of ponds every 2 days.

### 2.3 Physical and chemical analysis

Temperature, salinity, pH, conductivity, DO, and total dissolved solids (TDS) were estimated on site using a multiparameter probe (YSI Professional Plus; Yellow Springs, OH, United States). Surface water was collected in a 5-L water sampler for nutrient, bacterial, and plankton analyses. The volume required for nutrient analysis (3 L) was immediately filtered through a 0.45-µm microporous membrane and stored at −20°C for further analysis. The total nitrogen (TN), total phosphorus (TP), ammonium, nitrate, and nitrite concentrations were determined using standard methods (SEPAC, 1996), and the total organic carbon (TOC) was quantified using a TOC-L analyser (Shimadzu Corporation).

### 2.4 Amplicon sequencing of the bacteria

Surface water samples (200–500 mL) for microbial analysis were filtered through 0.22-μL pore-size polycarbonate filters (47 mm diameter; Millipore, Billerica, MA, USA). The membranes containing the microbes were stored at −80°C until DNA extraction (Liu et al., 2015). They were cut into small pieces with a sterilised cutter, and total DNA was extracted using a FastDNA SPIN Kit (MP Biomedicals, Santa Ana, CA, USA) following the manufacturer’s instructions. The DNA concentration was measured using a NanoDrop ND-8000 spectrophotometer (Thermo Scientific, Wilmington, DE, United States). High-throughput (next-generation) sequencing was used to characterise the composition and dynamics of bacteria.

The V4–V5 hypervariable regions of the bacterial 16S rRNA gene (130 bp) were amplified using the primers 515F (5′-CCA​TCT​CAT​CCC​TGC​GTG​TCT​CCG​ACT​CAG-3′) and 909R (5′- CCT​ATC​CCC​TGT​GTG​CCT​TGG​CAG​TCT​CAG-3′) (Tamaki et al., 2011). The samples were amplified in a reaction volume of 25 μL containing 12.50 μL of 2× GoTaq Master Mix (NEB, USA). The PCR parameters were as follows: initial denaturation at 95°C for 2 min; followed by 25 cycles at 95°C for 1 min, 56°C for 0.50 min, and 72°C for 1 min; and a final extension at 72°C for 10 min. Sequencing was performed on an Illumina MiSeq platform (Illumina, San Diego, California, United States) using a paired-end approach.

### 2.5 Identification and quantification of plankton

Surface water (1 L) was collected, preserved with Lugol’s solution, and allowed to stand undisturbed for 24 h. The supernatant was then removed, and the remaining sediment was poured into a 50-mL plastic bottle for further analysis of phytoplankton and rotifer abundances. Cladocerans and copepods were collected by filtering 20 L of the surface water through a 64-µm mesh zooplankton net, followed by preserving them with Lugol’s solution. All Lugol-preserved samples were stored in the dark at 4°C until analysis.

Phytoplankton samples were counted using 100-μL tubular chambers after thorough mixing in the sample flask to ensure that the subsampling represented the whole sample. Rotifers and crustaceans were counted in a plankton-counting chamber (1,000 μL and 5,000 μL chambers for rotifers and crustaceans, respectively) (CC-F, China). A minimum of 200 individuals for both the phytoplankton and zooplankton samples were counted from each sample. All plankton samples were identified to the species or genus level. The taxonomic identification of phytoplankton followed Hu and Wei. (2006). The zooplankton (rotifer, cladoceran, and copepod) identification followed Koste. (1978), Jiang and Du. (1979), and Sheng. (1979), respectively. The plankton in the samples were identified using fluorescence microscopy (BX-53; OLYMPUS, Japan).

### 2.6 Data analysis

To reveal temporal patterns in aquatic communities, a time-lag analysis was used to quantify the Bray–Curtis dissimilarity between each group of samples, and the time-lag was then plotted against the dissimilarity (Collins et al., 2000). Several general theoretical patterns can be deduced using this time-lag analysis method. If the sample distance increases over time, the community is unstable and undergoing a directional change. If the distance between samples does not change as the time-lag increases, the community is stable. If the sample distance decreases over time, the community is unstable and converging (Collins et al., 2000). The variation in the microbial community over time based on Bray–Curtis distance matrices was calculated using the “ggplot2” package in the R software (version 4.2.0). A histogram was used to illustrate the relative abundance of bacteria and was painted by the “RColorBrewer” package in R (version 4.2.0). Heatmaps of prokaryotic and eukaryotic communities were produced using the “pheatmap” package in R (version 4.2.0). Correlations between organisms were determined using the Spearman’s rank correlation test with the R package “psych”, and community networks of the significantly related species (r > 0.6, *p* < 0.01) were constructed and analysed using the R package “igraph” (Ju et al., 2014). Line and bar figures were plotted using Origin 2017.

## 3 Results and discussion

### 3.1 Environmental factors during the experiment

Water quality is a crucial factor affecting aquaculture systems, and its deterioration can result in significant costs for management (Wang et al., 2014). One of the objectives of the present study was to evaluate the application of microalgae as a method for water purification in rice-crayfish culture systems. Our results demonstrated that microalgal application can affect the concentrations of TDS, TN, and TOC in these systems. Mean value of TDS concentration in the MA group was 1.01-fold higher than that in the no-MA group, whereas the mean TN concentration was much lower in the MA group during the experimental period. Furthermore, the mean TOC concentration in the MA group was higher than that in the no-MA group (Table 1). Mean values of NOx-N (sum of nitrate and nitrite) and NH_4_^+^-N concentration in the MA group were lower than in the no-MA group (Table 1), consistent with the trend observed for TN. Water temperature, DO, salinity, pH and TP concentration did not differ significantly between the two groups (Table 1). Higher TDS and TOC contents suggest that microbial density was higher in the microalgal treatment group (Nam et al., 2021; Chen et al., 2022). Nitrogen is an important factor for aquacultural production (Smith and Piedrahita, 1988; Wang et al., 2021; Jiao et al., 2022), and its effects are discussed below.

**TABLE 1:** Environmental factors (mean values ±standard deviation) in crayfish culture system under different treatment conditions and differences between groups.

	WT (°C)	DO (mg L^-1^)	TDS (mg L^-1^)	Salinity	pH	TOC (mg L^-1^)	TN (mg L^-1^)	TP (mg L^-1^)	NOx-N (mg L^-1^)	NH_4_^+^-N (mg L^-1^)
**No-MA group**	25.54 ± 2.72	8.04 ± 1.64	279 ± 24	0.21 ± 0.02	8.04 ± 0.30	8.81 ± 1.44	5.02 ± 0.31	0.16 ± 0.02	1.34 ± 0.14	1.48 ± 0.44
**MA group**	25.93 ± 2.78	8.43 ± 1.26	283 ± 22	0.21 ± 0.02	8.03 ± 0.13	10.28 ± 4.67	4.55 ± 0.23	0.15 ± 0.01	1.20 ± 0.02	1.19 ± 0.05

No-MA, group: the group without microalgal application; MA, group: the group with microalgal application; WT, water temperature; DO, dissolved oxygen; TDS, total dissolved solid; TOC, total organic carbon; TN, total nitrogen; TP, total phosphorus; NOx-N, sum of nitrate and nitrite; NH_4_^+^-N: content of ammonia nitrogen.

**FIGURE 1:**
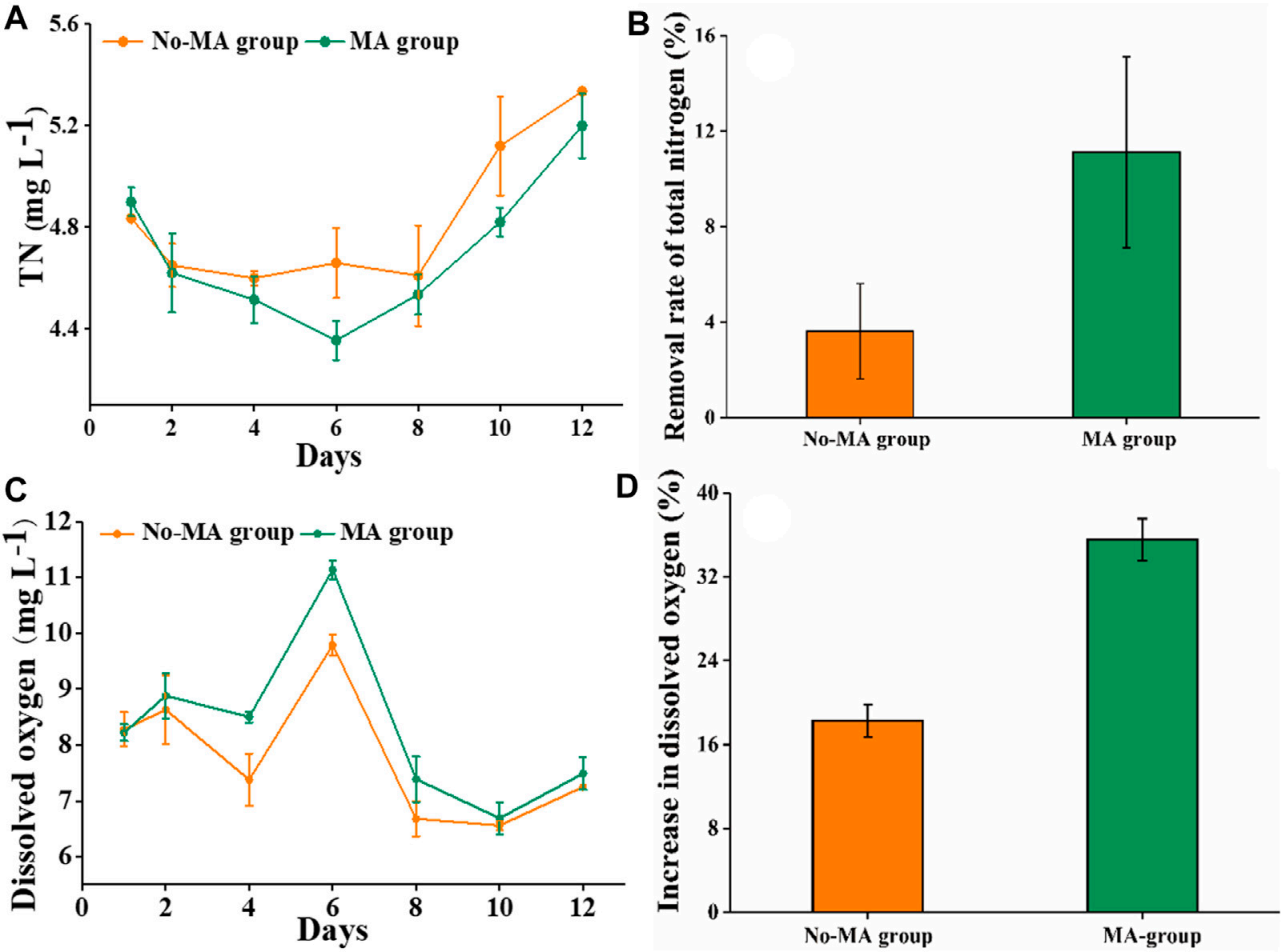
Total nitrogen (TN) and dissolved oxygen (DO) in the microalgae (MA) and no microalgae (No-MA) application groups, where **(A)** is the temporal dynamics of TN, **(B)** is the removal rate of TN on day 6, **(C)** is the temporal dynamics of DO, and **(D)** is the proportional increase of DO on day 6 compared with the initial value. Error bars indicate ±standard deviation (SD).

The TN content showed a moderate change in the absence of microalgae, with a gradual decrease following the application of microalgae. However, the TN content in both groups showed a gradual and consistent increase after day 6. This may be due to the growth of microalgae was not sufficient to further reduce the nitrogen content generated by residual feed, feces, and other organic matter after 6 days. However, it is possible that the addition of microalgae again could stimulate continued nitrogen utilization. On day 6, the difference in the TN content between the two groups was the greatest (Figure 1A). In addition, TN removal rates on the 6th day were 3.6% and 11.1% in the no-MA and MA groups, respectively (Figure 1B). High nutrient concentrations can cause eutrophication in receiving water bodies, leading to rapid growth of filamentous algae and impeding crayfish growth (Sauthier et al., 1998). We also assessed DO levels, given their importance in aquacultural production. Although overall DO levels did not differ significantly between the two groups, DO content in the MA group was higher than that in the no-MA group during the first 6 days (Figure 1C), with the largest difference observed on day 6 (Figure 1D). Collectively, these findings demonstrate that microalgal application can enhance the water quality of crayfish aquaculture systems by effectively reducing total nitrogen content and increasing dissolved oxygen content to some extent, with the most significant effect observed on the sixth day after microalgal application.

### 3.2 Characteristics of bacterial community structure

**FIGURE 2:**
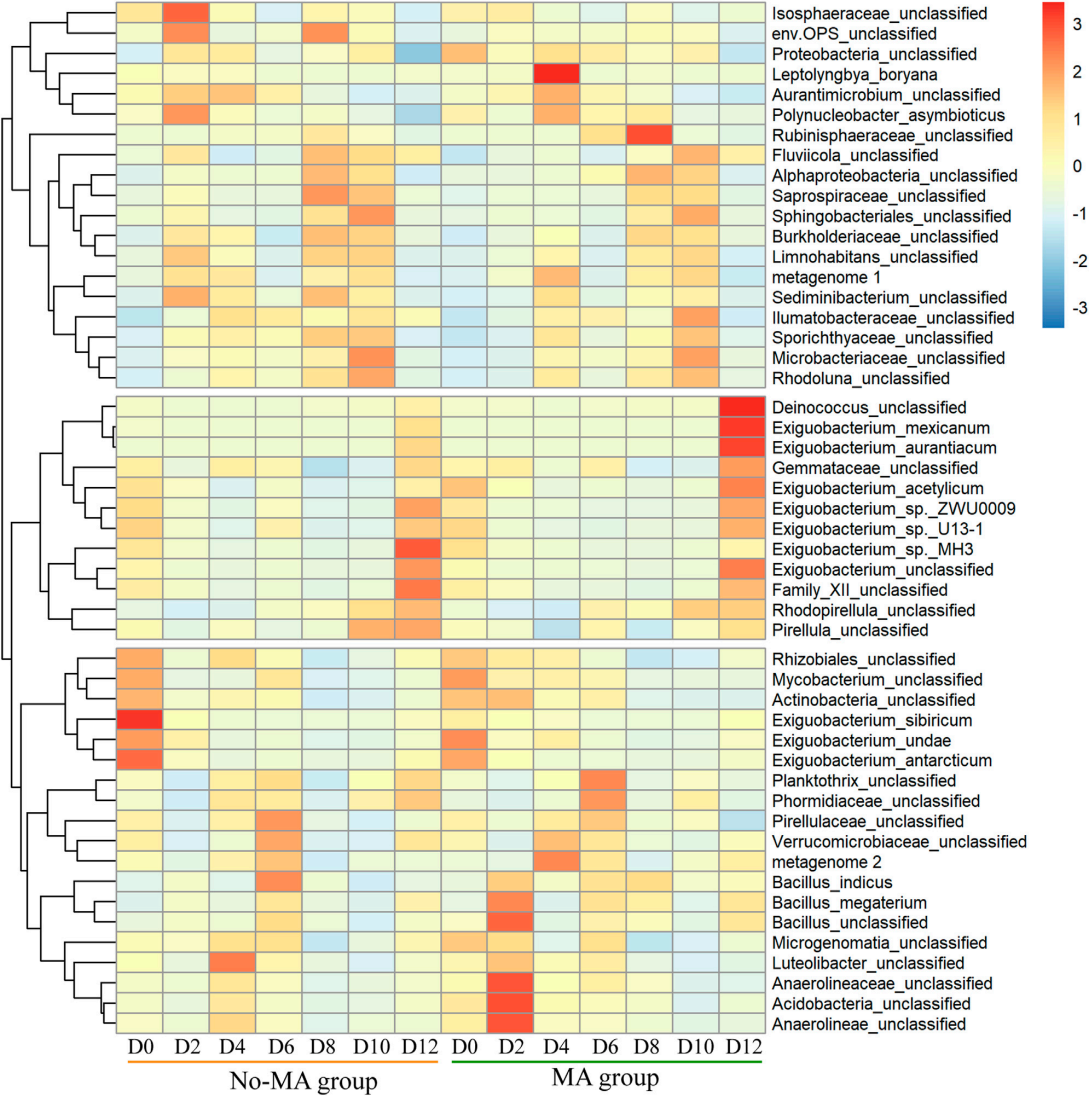
Temporal variation in the relative abundance of bacteria (top 50) from the initial day (D0) to day 12 (D12) in the microalgae (MA) and no microalgae (No-MA) application groups.

The species composition of the bacterial community under the microalgal application was similar to that no-MA group at the phylum level (Supplementary Figure S1), but species abundance differed between the two groups. The abundances of *Deinococcus*, *Exiguobacterium*, and Gemmataceae were higher under the microalgal application at the end of the experiment (Figure 2). The genus *Deinococcus* has been isolated worldwide, and its presence in an aquatic ecosystem can indicate a nutrient-poor environment (White et al., 1999). *Exiguobacterium* species possess unique properties of interest for applications in biotechnology, bioremediation, industry, and agriculture (Zhang et al., 2013). Among them, *E. mexicanum* and *E. aurantiacum* are nitrate reductase positive, suggesting that they can produce energy by reducing nitrate (NO_3_^−^) to nitrite (NO_2_^−^) or nitrogen (N_2_) (Vishnivetskaya et al., 2009). In contrast, *E. acetylicum* is oxidase positive and can therefore utilise oxygen for energy production using an electron transfer chain (Vishnivetskaya et al., 2009). Therefore, the higher abundance of *Exiguobacterium* species detected in the MA group in the present study proves that microalgal addition contributed to the utilisation of N and promoted a more aerobic environment. In addition, according to Guo et al. (2019), *Exiguobacterium* is a crucial group of intestinal microbes for *P. clarkii*. This suggests that the addition of microalgae may enhance the metabolism of *P. clarkii*. Gemmataceae is a large family, most members of which are found in various aquatic habitats (Ivanova et al., 2021), but the significance of the presence of Gemmataceae in the present study is unclear and should be investigated further.

**FIGURE 3:**
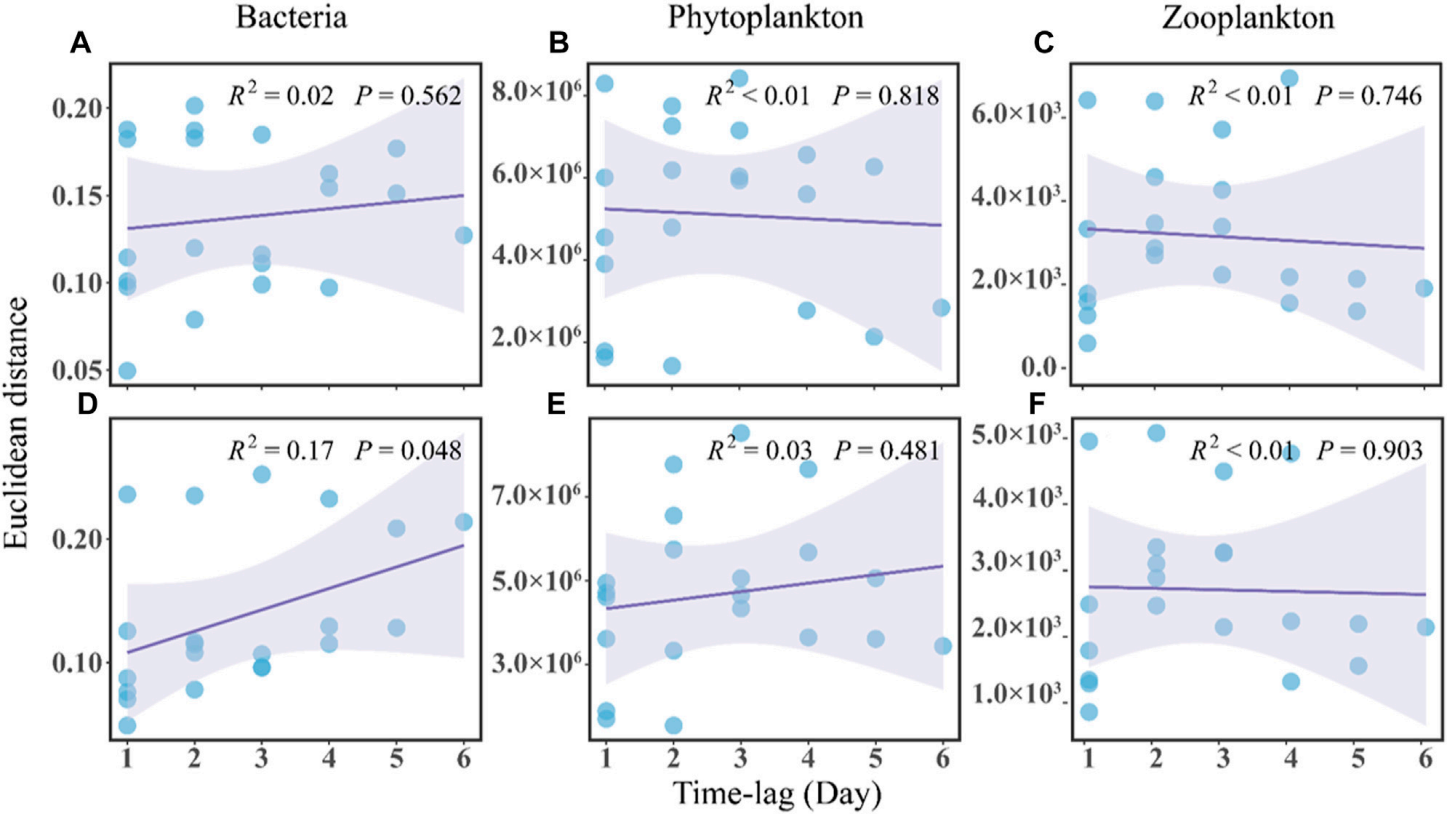
Time-lag regression analysis of the microbial communities in the no microalgae (No-MA) **(A–C)** represent bacteria, phytoplankton, and zooplankton, respectively and microalgae (MA) **(D–F)** represent bacteria, phytoplankton, and zooplankton, respectively application groups.

In terms of community stability, the regression line slope for the no-MA group was not significantly different from zero and the overall regression was not significant, indicating a stochastic variation of the bacterial community over time (Figure 3A). The slope for the MA group was positive, implying a directional change in the bacterial community (Figure 3D). This along with the greater abundance of *Deinococcus* and *Exiguobacterium* in the MA group may suggest an ecological and functional shift towards a more oligotrophic ecology with microalgal application.

### 3.3 Characteristics of plankton community structure

**FIGURE 4:**
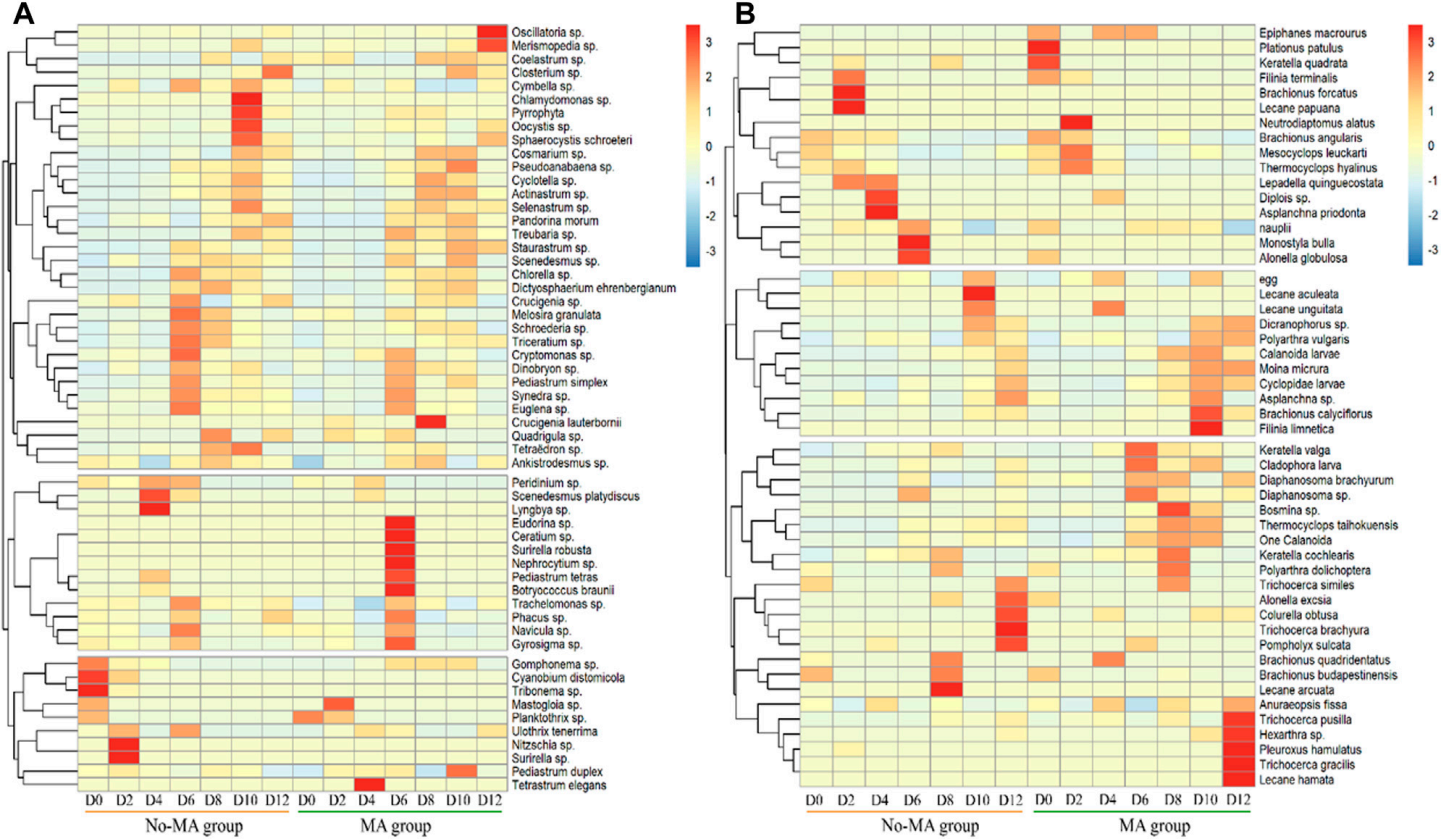
Temporal variation of the relative abundance of plankton **(A)** for phytoplankton, **(B)** for zooplankton at the genus and species level from the initial day (D0) to day 12 (D12) in the microalgae (MA) and no microalgae (No-MA) application groups.

The plankton species composition under the microalgal application was similar to that no-MA group at the phylum level (Supplementary Figures S2, S3), but species abundance differed between the two groups. There was a shift in the phytoplankton population on day 6 (Supplementary Figure S2). During the first part of the experiment (until day 6), one chlorophyte (*Scenedesmus platydiscus*), two cyanophytes (*Lyngbya* sp. and *Cyanobium distomicola*), one xanthophyte (*Tribonema* sp.), and two bacillariophytes (*Nitzschia* sp. and *Surirella* sp.) were the main phytoplankton in the no-MA group and one bacillariophyte (*Mastogloia* sp.) and one chlorophyte (*Tetrastrum elegans*) were the main phytoplankton in the MA group. After day 6, three chlorophytes (*Chlamydomonas* sp., *Oocystis* sp., and *Sphaerocystis schroeteri*) and one pyrrophyte were dominant in the no-MA group and two cyanophytes (*Oscillatoria* sp. and *Merismopedia* sp.), five chlorophytes (*Crucigenia lauterbornii*, *Eudorina* sp., *Nephrocytium* sp., *Pediastrum tetras*, and *Botryococcus braunii*), one pyrrophyte (*Ceratium* sp.), and one bacillariophyte (*Surirella robusta*) were dominant in the MA group (Figure 4A).

The results showed that the dominant species of phytoplankton decreased in the no-MA group, and those of the MA group increased during the process of crayfish cultivation. Zhang et al. (2021) noted that a diverse phytoplankton community can help maintain water stabilisation, indicating that microalgal application will contribute to aquaculture stability. There was no significant increase in the number of *Scenedesmus* cells in the MA group. Zhang et al. (2021) stated that heterotrophic cells of *Scenedesmus* were significantly heavier and larger than photoautotrophic cells. Therefore, the heterotrophic cells of *Scenedesmus* were more likely to sink to the pond bottom, but they could still absorb nutrients and grow after sinking.

The shift in the zooplankton population occurred on day 8 (Supplementary Figure S3). During the first 8 days of the experiment, five rotifers (*Brachionus falcatus*, *Lecane papuana*, *Diplois* sp., *Asplanchna priodonta*, and *Monostyla bulla*) and one cladoceran (*Alonella globulosa*) were predominant in the no-MA group and two rotifers (*Plationus patulus* and *Keratella quadrata*) and one copepod (*Neutrodiaptomus alatus*) were predominant in the MA group. After day 8, three rotifers (*Lecane arcuate*, *Lecane aculeate*, and *Trichocerca brachyura*) were dominant in the no-MA group and five rotifers (*Filinia limnetica*, *Trichocerca pusilla*, *Hexarthra* sp., *Trichocerca gracilis*, and *Lecane hamata*) and one cladoceran (*Pleuroxus hamulatus*) were dominant in the MA group (Figure 4B). The results showed that the dominant species of zooplankton decreased in the no-MA group, and those of the MA group increased under microalgal application. This indicated that microalgal application contributed to zooplankton diversity, which is important to system stability (Zhang et al., 2022).

Analysis of the plankton community structure in the present study showed an insignificant convergence trend in the no-MA group (Figures 3B,C) and no changes in the MA group (Figures 3E,F). It can be concluded that microalgal application in the crayfish system has a certain impact on the structure of the plankton community in comparison to the system without microalgae. Compared with the structure of the bacterial community, the plankton structure did not change much with microalgal application, which may be because of the lower sensitivity of planktonic organisms to environmental variations. Jiao et al. (2005) pointed out that bacterial community structures change faster than plankton community structures in response to environmental variations. In addition, plankton are a natural feed for crayfish. However, no significant difference in the fatty acid composition of *P. clarkii* found between the MA group and no-MA group (Supplementary Figure S4). A potential reason for the negligible influence of microalgae addition on crayfish growth is that crayfish tend to selectively consume the bait in order to gain maximum net energy, which is the energy obtained from food minus the energy expended in processing it. This is consistent with the study of Li et al. (2019), who found that microalgae performed well at removing nutrients, but the plankton biomass was far from enough to feed the oyster that was being cultivated. In summary, the study showed that microalgal application had limited effect on plankton community structure and no effect on *P*. *clarkii* growth.

### 3.4 Variation in *Spirogyra* abundances in the crayfish ponds

**FIGURE 5:**
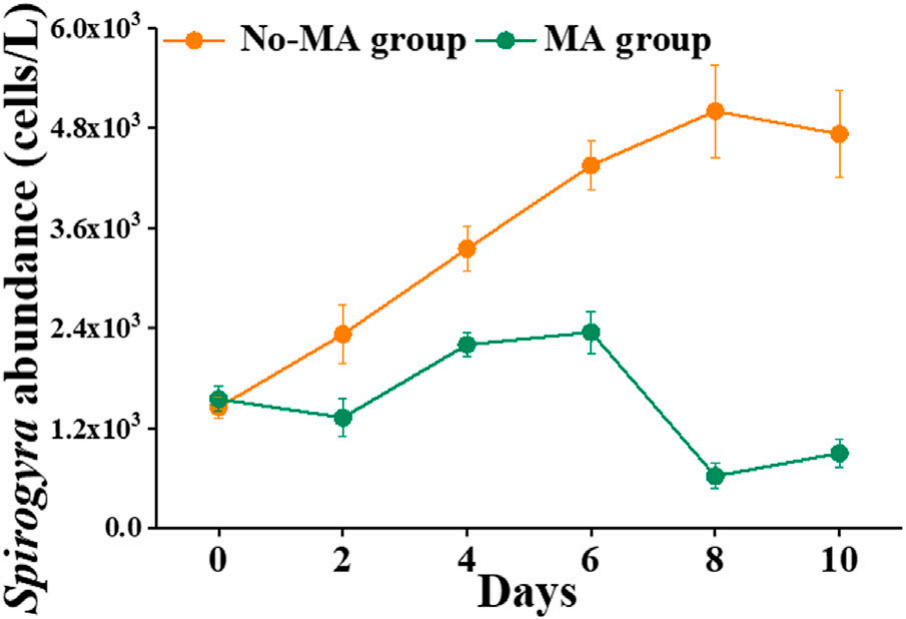
Temporal variation in *Spirogyra* abundance in the microalgae (MA) and no microalgae (No-MA) application groups.

Another difference between the no-MA and MA groups was that the abundance of *Spirogyra* was higher in the no-MA group than in the MA group (Figure 5). Over time, *Spirogyra* abundance increased gradually without microalgal application but not under the microalgal application (Figure 5). In the no-MA group, *Spirogyra* abundance increased from 1,450 cells L^−1^ to 5,000 cells L^−1^ (the highest number recorded) on day 8. In contrast, *Spirogyra* abundance decreased from 1,550 cells L^−1^ to 625 cells L^−1^ (the lowest number recorded) in the MA group on day 8. *Spirogyra* abundance increased by 225.9% in the no-MA group and decreased by 41.9% in the MA group compared with its initial value. The number of *Spirogyra* in the MA group was nearly five times less than that no-MA group.

Higher numbers of *Spirogyra* spp. are detrimental for crayfish cultivation. Ulikowski et al. (2015) proved that the presence of filamentous algae (*Spirogyra*) has a strong negative impact on juvenile crayfish survival. It is clear from the present study that microalgal addition can significantly change the water quality of crayfish systems to inhibit the growth of filamentous algae such as *Spirogyra*. Moreover, low concentration of nutrients can inhibit the rapid growth of filamentous algae (Havens et al., 1999). This is consistent with the observation that the abundance of *Spirogyra* under the microalgal application, which had lower TN concentration (Table 1), was significantly lower than that without microalgal application (Figure 5). This illustrates how the manipulation of one set of microorganisms can influence environmental factors that in turn affect the growth of other microorganisms (Nam et al., 2021). This is also consistent with the assumption that variations in the aquatic environment in the water column are always coupled with changes in microorganism community structure (Zhang et al., 2021). In conclusion, microalgal application can inhibit growth of the harmful algae *Spirogyra* by absorbing nitrogen.

### 3.5 Network analysis of microorganisms in crayfish culture

**FIGURE 6:**
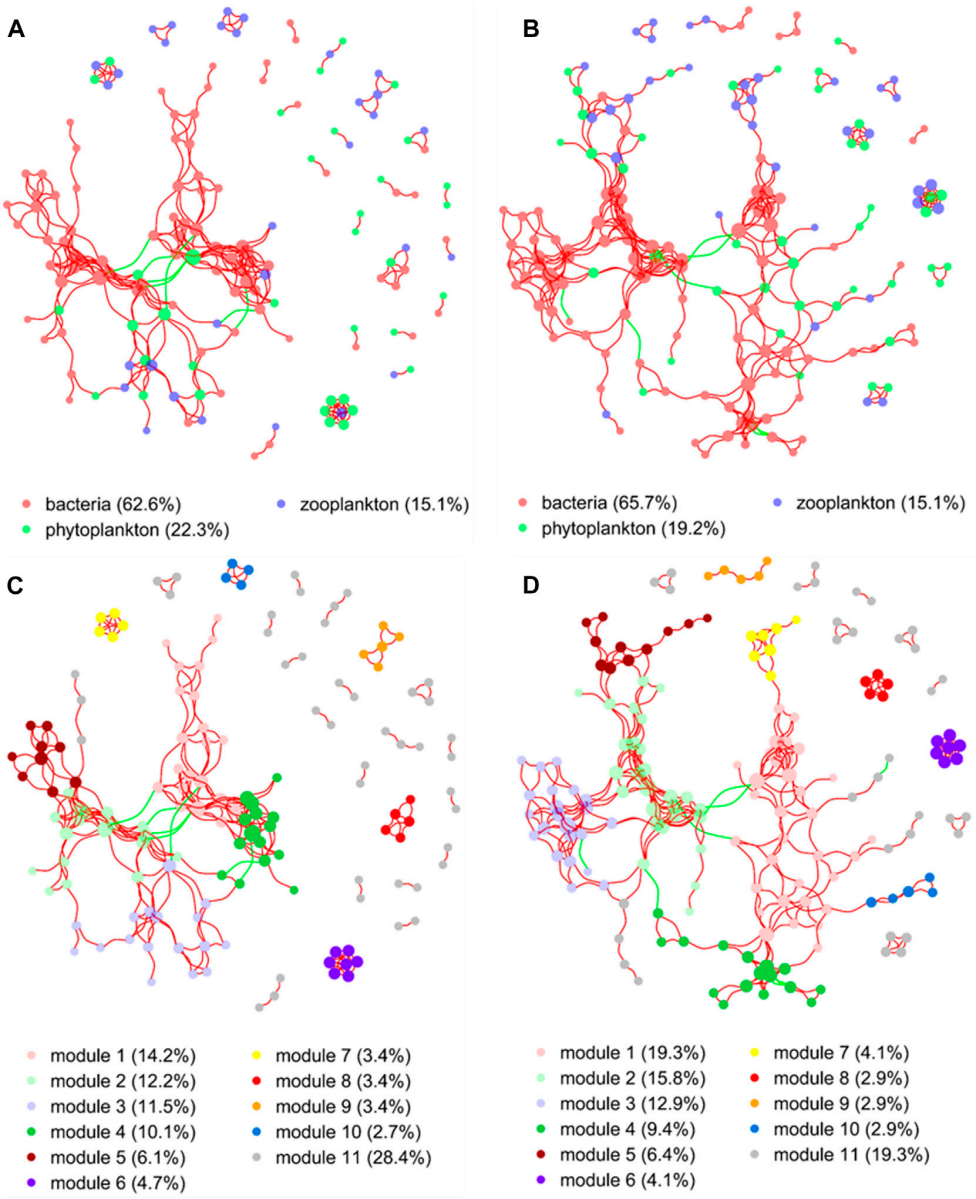
Co-occurrence network analysis demonstrating the associations between microorganisms in the no microalgae (No-MA) **(A,C)** and microalgae (MA) **(B,D)** application groups. A connection represents a strong (Spearman’s r > 0.60) and significant (*p* < 0.01) correlation (red colour represents a positive interaction; green colour represents a negative interaction). Nodes are coloured according to different taxonomic categories **(A,B)** and modules **(C,D)**. The size of each node is proportional to the number of connections. Percentages are the percentage of total nodes, with modules listed in decreasing percentage order except for others, which represents the remainder of the nodes.

The network structure of microorganisms clearly differed between the two groups in the present study. The clustering coefficient (0.56) and modularity (0.77) of the MA group were higher than those of the no-MA group (0.54 and 0.74, respectively), indicating that the microorganism community network in the MA group had modular structures and small-world properties—that is, it had high interconnectivity (Table 2). In the no-MA group, the network consisted of 148 nodes (representing genera) linked by 298 edges, which were mainly positively correlated (Table 2; Figure 6). Bacteria (62.6%) comprised the predominant group in the network (Figure 6A), and the percentages represented by modules 1–4 were similar, ranging from 10.1% to 14.2% (Figure 6C). In the MA group, the network consisted of 171 nodes and 372 edges (Table 2), with more positive than negative correlations. Bacteria (65.7%) were the predominant group in the network (Figure 6B). Modules 1 (19.3%), 2 (15.8%), and 3 (12.9%), the top three microorganism community modules in the MA group, were significantly positively correlated with each other (Figure 6D).

**TABLE 2:** Topological properties of the species–species co-occurrence networks of microorganism communities in the microalgae (MA) and no microalgae (No-MA) application groups.

Groups	Number of edges	Number of nodes	Average path length	Network density	Clustering coefficient	Modularity
**No-MA group**	298	148	4.13	0.11	0.54	0.74
**MA group**	372	171	6.10	0.11	0.56	0.77

Network analysis revealed a higher modularity, interconnectivity, and complexity of the microorganism community in the MA group than the no-MA group. This suggests that the microorganism network structure are inherently different, even when the diversity and composition of the microorganism communities are comparable (Zhao et al., 2016). Ecologists believe that the increasing complexity of trophic relationships increases ecosystem stability (McCann et al., 1998; Pan et al., 2021; Priyadarshi et al., 2022), which is particularly important in the maintenance of a healthy and sustainable aquaculture system. These network analysis results along with the fact that changes in TN and DO after microalgal addition promoted the occurrence of denitrifying and aerobic bacteria may indicate that the microalgal application first changes the water quality characteristics in the crayfish system, which is followed by changes to the microorganism community structure, making it more stable.

Despite the clear increase in the stability of the crayfish systems after microalgal application in this study, this increase may be brief, since the interactions among organisms were mainly positively correlated and bacteria played a dominant role in the network structure. This indicates that variation in the bacterial community structure would eventually affect the composition of the plankton community. The change in plankton community was not significant in this study, but this may be attributed to the fact that the microalga was added only once in this experiment, and perhaps further additions are needed for the treatment to work effectively. Moreover, the difference in TN and DO between the no-MA and MA groups was maximal on day 6 after microalgal addition. Therefore, microalgae should be added to crayfish systems periodically, at intervals of 6 days, to maximise the effect of bioremediation. In summary, the co-occurrence patterns revealed through network analysis in this study offered a new insight into the potential interactions between microorganisms in crayfish cultivation systems. This in turn provides a better understanding of the microorganism community structures in such systems, which will help in the formation of a theoretical basis for the provision of better guidance on water quality control.

## 4 Conclusion

Microalgae is a very promising organism to be used in aquaculture. Microalgae can improve the water quality of crayfish systems by removing TN efficiently. Moreover, microalgal addition can cause a directional change in the bacterial community structure, inhibit the growth of filamentous algae (*Spirogyra*) by 81.0%, and moderately affect the plankton community, thereby forming a stable ecosystem that contributes to the development of a sustainable industrial rearing system. The purification effect of microalgae reached the maximum on the 6^th^ day, so we speculated that microalgae should be added to crayfish systems periodically (every 6 days) to maximise the effect of bioremediation. Even if the present findings may reflect some advantages of microalgae using in the rice-crayfish system, studies on prolonged and/or repeated use of microalgae are necessary to derive the general benefits.

## Data Availability

The original contributions presented in the study are included in the article/Supplementary Material, further inquiries can be directed to the corresponding authors.

## References

[B1] Alcorlo P. Geiger W. Otero M. (2004). Feeding preferences and food selection of the red swamp crayfish, *Procambarus clarkii*, in habitats differing in food item diversity. Crustaceana 77 (4), 435–453. 10.1163/1568540041643283

[B2] Chen W. Wang Y. Wang L. Ji Y. Wang Q. Li M. (2022). Emerging investigator series: Effects of sediment particle size on the spatial distributions of contaminants and bacterial communities in the reservoir sediments. Environ. sci-wat Res. 8, 957–967. 10.1039/D1EW00877C

[B3] Collins S. L. Micheli F. Hartt L. (2000). A method to determine rates and patterns of variability in ecological communities. Oikos 91, 285–293. 10.1034/j.1600-0706.2000.910209.x

[B4] Gao R. J. Chen L. Zhang W. Zhang S. Rao J. Hu J. (2020). Effect of dietary Antarctic krill euphausia superba on the growth performance and nonspecific immunity of red swamp crayfish *Procambarus clarkia* . Fish. Shellfish Immunol. 96, 122–125. 10.1016/j.fsi.2019.12.004 31805411

[B5] Garnier A. Hulot F. D. Petchey O. L. (2020). Manipulating the strength of organism-environment feedback increases nonlinearity and apparent hysteresis of ecosystem response to environmental change. Ecol. Evol. 1, 5527–5543. 10.1002/ece3.6294 PMC7319241 32607172

[B6] Guo K. Ruan G. Fan W. Fang L. Wang Q. Luo M. (2019). The effect of nitrite and sulfide on the antioxidant capacity and microbial composition of the intestines of red swamp crayfish, *Procambarus clarkia* . Fish. Shellfish Immunol. 96, 290–296. 10.1016/j.fsi.2019.11.052 31765791

[B7] Havens K. E. East T. L. Hwang S. J. Rodusky A. J. Sharfstein B. Steinman A. D. (1999). Algal responses to experimental nutrient addition in the littoral community of a subtropical lake. Freshw. Biol. 42, 329–344. 10.1046/j.1365-2427.1999.444479.x

[B8] Hu H. J. Wei Y. X. (2006). Freshwater algae in China systematics, classification and ecology. Beijing: Science Press.

[B9] Ivanova A. A. Miroshnikov K. K. Oshkin I. Y. (2021). Exploring antibiotic susceptibility, resistome and mobilome structure of planctomycetes from *Gemmataceae* family. Sustainability 13, 5031. 10.3390/su13095031

[B10] Jiang S. C. Du N. S. (1979). Fauna sinica, Crustacea, freshwater cladocera. Beijing: Science Press.

[B11] Jiao N. Z. Yang Y. H. Hong N. Ma Y. Harada S. Koshikawa H. (2005). Dynamics of autotrophic picoplankton and heterotrophic bacteria in the East China Sea. Cont. Shelf Res. 25, 1265–1279. 10.1016/j.csr.2005.01.002

[B12] Jiao Y. Zhao H. Li Z. Tang X. Li Y. Chen S. (2022). Nitrogen budgets for freshwater aquaculture and mariculture in a large tropical island – a case study for Hainan Island 1998–2018. Environ. Res. 177, 105642. 10.1016/j.marenvres.2022.105642 35567873

[B13] Jin H. Zhang H. Zhou Z. W. Li K. P. Hou G. L. Xu Q. (2020). Ultrahigh‐cell‐density heterotrophic cultivation of the unicellular green microalga *Scenedesmus acuminatus* and application of the cells to photoautotrophic culture enhance biomass and lipid production. Biotechnol. Bioeng. 117, 96–108. 10.1002/bit.27190 31612991 PMC6916281

[B14] Ju F. Xia Y. Guo F. Wang Z. Zhang T. (2014). Taxonomic relatedness shapes bacterial assembly in activated sludge of globally distributed wastewater treatment plants. Environ. Microbiol. 16, 2421–2432. 10.1111/1462-2920.12355 24329969

[B15] Koste W. (1978). Rotatoria, die rä dertiere mitteleuropas. Ein bestimmungswerk, begründet von Max voigt. Ü berordnung monogononta 2. Berlin: Gebrüder Borntraeger.

[B16] Kumar S. D. Santhanam P. Min S. P. Kim M. K. (2016). Development and application of a novel immobilized marine microalgae biofilter system for the treatment of shrimp culture effluent. J. Water Process. 13, 137–142. 10.1016/j.jwpe.2016.08.014

[B17] Li M. Callier M. D. Blancheton J. P. Galès A. Nahon S. T. Geoffroy T. (2019). Bioremediation of fishpond effluent and production of microalgae for an oyster farm in an innovative recirculating integrated multi-trophic aquaculture system. Aquaculture 504, 314–325. 10.1016/j.aquaculture.2019.02.013

[B18] Liu F. Shao G. Y. Tian Q. Q. Cheng B. X. Yu Y. B. Wang A. M. (2021). Enhanced growth performance, immune responses, immune-related gene expression and disease resistance of red swamp crayfish (*Procambarus clarkii*) fed dietary glycyrrhizic acid. Aquaculture 533, 736202. 10.1016/j.aquaculture.2020.736202

[B19] Liu L. M. Yang J. Yu Z. Wilkinson D. M. (2015). The biogeography of abundant and rare bacterioplankton in the lakes and reservoirs of China. ISME J. 9, 2068–2077. 10.1038/ismej.2015.29 25748371 PMC4542038

[B20] Lu Q. Yang L. Deng X. (2020). Critical thoughts on the application of microalgae in aquaculture industry. Aquaculture 528, 735538. 10.1016/j.aquaculture.2020.735538

[B21] McCann K. Hastings A. Huxel G. R. (1998). Weak trophic interactions and the balance of nature. Nature 395, 794–798. 10.1038/27427

[B22] Naiman R. J. Elliott S. R. Helfield J. M. O’Keefe T. C. (1999). Biophysical interactions and the structure and dynamics of riverine ecosystems: The importance of biotic feedbacks. Hydrobiologia 410, 79–86. 10.1007/978-94-017-2163-9_9

[B23] Nam S. Alday J. G. Kim M. Kim H. Ji Y. J. Park T. (2021). The relationships of present vegetation, bacteria, and soil properties with soil organic matter characteristics in moist acidic tundra in Alaska. Sci. Total Environ. 772, 145386. 10.1016/j.scitotenv.2021.145386 33770858

[B24] Oliveira C. Y. B. Oliveira C. D. L. Prasad R. Ong H. C. Evando S. A. Shabnam N. (2021). A multidisciplinary review of *Tetradesmus obliquus*: A microalga suitable for large-scale biomass production and emerging environmental applications. Rev. Aquac. 13, 1594–1618. 10.1111/raq.12536

[B25] Pan A. Chen Y. H. Zhou M. McAllister T. A. Guan L. L. (2021). Microbial interaction-driven community differences as revealed by network analysis. Comput. Struct. Biotechnol. J. 19, 6000–6008. 10.1016/j.csbj.2021.10.035 34849204 PMC8599104

[B26] Priyadarshi A. Chandra R. Kishi M. J. Smith S. L. Yamazaki H. (2022). Understanding plankton ecosystem dynamics under realistic micro-scale variability requires modeling at least three trophic levels. Ecol. Modell. 467, 109936. 10.1016/j.ecolmodel.2022.109936

[B27] Ramli N. M. Yusoff F. M. Giatsis C. Tan G. Verdegem M. (2018). Effects of *Stigeoclonium nanum*, a freshwater periphytic microalga on water quality in a small-scale recirculating aquaculture system. Aquac. Res. 49, 1–12. 10.1111/are.13818

[B28] Sauthier N. Grasmick A. Blancheton J. P. (1998). Biological denitrification applied to a marine closed aquaculture system. Water Res. 32, 1932–1938. 10.1016/S0043-1354(97)00406-5

[B29] Sheng J. R. (1979). Fauna sinica, Crustacea, freshwater copepoda. Beijing: Science Press.

[B30] Smith D. W. Piedrahita R. H. (1988). The relation between phytoplankton and dissolved oxygen in fish ponds. Aquaculture 68, 249–265. 10.1016/0044-8486(88)90357-2

[B31] State Environmental Protection Administration of China (SEPAC) (1996). Water and exhausted water monitoring analysis method. Beijing: Chinese Environmental Press.

[B32] Tamaki H. Wright C. L. Li X. Lin Q. Hwang C. Wang S. (2011). Analysis of 16S rRNA amplicon sequencing options on the Roche/454 next-generation titanium sequencing platform. PLoS One 6, e25263. 10.1371/journal.pone.0025263 21966473 PMC3179495

[B33] Tejido-Nunez Y. Aymerich E. Sancho L. Refardt D. (2019). Treatment of aquaculture effluent with *Chlorella vulgaris* and *Tetradesmus obliquus*: The effect of pretreatment on microalgae growth and nutrient removal efficiency. Ecol. Eng. 136, 1–9. 10.1016/j.ecoleng.2019.05.021

[B34] Ulikowski D. Chybowski L. Traczuk P. (2015). Harmful impact of filamentous algae (*Spirogyra* sp) on juvenile crayfish. Arch. Pol. Fish. 23, 223–226. 10.1515/aopf-2015-0025

[B35] Vishnivetskaya T. Kathariou S. Tiedje J. M. (2009). The *Exiguobacterium* genus: Biodiversity and biogeography. Extremophiles 13, 541–555. 10.1007/s00792-009-0243-5 19381755

[B36] Wang C. Jiang C. C. Gao T. M. Peng X. W. Ma A. L. Sun Q. (2021). Improvement of fish production and water quality in a recirculating aquaculture pond enhanced with bacteria-microalgae association. Aquaculture 547, 737420–737513. 10.1016/j.aquaculture.2021.737420

[B37] Wang Q. D. Cheng L. Liu J. S. Li Z. J. Xie S. Q. De Silva S. S. (2014). Freshwater aquaculture in PR China: Trends and prospects. Rev. Aquac. 5, 283–302. 10.1111/raq.12086

[B38] White Q. Eisen J. A. Heidelberg J. F. Hickey E. K. Peterson J. D. Dodson R. J. (1999). Genome sequence of the radioresistant bacterium *Deinococcus radiodurans* R1. Science 286, 1571–1577. 10.1126/science.286.5444.1571 10567266 PMC4147723

[B39] Yearbook C. F. S. (2022). China fishery statistic yearbook. Beijing: China Agriculture Press.

[B40] Zhang F. Man Y. B. Mo W. Y. Wong M. H. (2019). Application of *Spirulina* in aquaculture: A review on wastewater treatment and fish growth. Rev. Aquac. 12, 582–599. 10.1111/raq.12341

[B41] Zhang H. Zhao L. Chen Y. Zhu M. M. Xu Q. Wu M. C. (2021b). Trophic transition enhanced biomass and lipid production of the unicellular green alga *Scenedesmus acuminatus* . Front. Bioeng. Biotechnol. 9, 638726. 10.3389/fbioe.2021.638726 34095093 PMC8176925

[B42] Zhang K. Peng H. H. Xia Y. Gong W. B. Li Z. F. Yu E. M. (2022). Evaluating ecological mechanisms and optimization strategy of rice–fish co–culture system by ecosystem approach. Aquaculture 560, 738561. 10.1016/j.aquaculture.2022.738561

[B43] Zhang M. Dong J. Gao Y. N. Liu Y. Zhou C. J. Meng X. L. (2021a). Patterns of phytoplankton community structure and diversity in aquaculture ponds, Henan, China. Aquaculture 544, 737078. 10.1016/j.aquaculture.2021.737078

[B44] Zhang Y. Shi P. Ma J. (2013). *Exiguobacterium* spp. and their applications in environmental remediation. J. Environ. Biol. 19, 898–904. 10.3724/sp.j.1145.2013.00898

[B45] Zhao D. Feng S. Jin Z. Rui H. Yu Z. Wu Q. L. (2016). Network analysis reveals seasonal variation of co-occurrence correlations between cyanobacteria and other bacterioplankton. Sci. Total Environ. 573, 817–825. 10.1016/j.scitotenv.2016.08.150 27595939

