# Supplementary material for: Application of microalgae Scenedesmus acuminatus enhances water quality in rice-crayfish culture
Source: Front Bioeng Biotechnol. 2023 May 4;11:1143622. doi: 10.3389/fbioe.2023.1143622 (PMC10192885; doi:10.3389/fbioe.2023.1143622)
Supplement: Supplementary file 1 [file DataSheet1.docx]

**Microalgae improves water quality in rice-crayfish culture**

Danni Yuan^a,b^, Lan Wang^b,c*^, Hongxia Wang^b,c^, Rongli Miao^d^, Yulu Wang^b,c^, Hu, Jin^b^, Lu Tan^e^, Chaojun Wei^d^, Qiang Hu^f,g^, Yingchun Gong^b,c*^

a School of Environmental Ecology and Biological Engineering, Wuhan Institute of Technology, Wuhan 430205, PR China

b Center for Microalgal Biotechnology and Biofuels, Institute of Hydrobiology, Chinese Academy of Sciences, Wuhan 430072, PR China

c State Key Laboratory of Freshwater Ecology and Biotechnology, Institute of Hydrobiology, Chinese Academy of Sciences, Wuhan 430072, PR China

d Hydrobiological Data Analysis Center, Institute of Hydrobiology, Chinese Academy of Sciences, Wuhan 430072, PR China

e Systems Ecology and Watershed Ecology Center for Freshwater Ecology, Institute of Hydrobiology, Chinese Academy of Sciences, Wuhan 430072, PR China

f CAS Key Laboratory of Quantitative Engineering Biology, Shenzhen Institute of Synthetic Biology, Shenzhen Institute of Advanced Technology, Chinese Academy of Sciences, Shenzhen 518055, PR China

g Faculty of Synthetic Biology, Shenzhen Institute of Advanced Technology, Chinese Academy of Sciences, Shenzhen 518055, PR China

* Corresponding author: Yingchun Gong, [springgong@ihb.ac.cn](mailto:springgong@ihb.ac.cn); Lan Wang, [wanglan@ihb.ac.cn](mailto:wanglan@ihb.ac.cn)

**1. Lipid extraction and fatty acid analysis**

Total lipids of Procambarus clarkii powders were extracted and measured using a modified Bligh and Dyer method that was described in Yang et al. (2019). Briefly, 50 mg of freeze-dried P. clarkia meat was ground into fine powder with liquid nitrogen and transferred to a labeled glass vial. 10 mL of chloroform and methanol (2:1, v/v) mixture as extraction solvent was added into the vial and the mixture was kept stirring at 130 rpm for 1 h in a thermal incubator. Then, 2.5 mL of 0.7% (w/v) potassium chloride was added into the vial and vortexed for 30 s, followed by centrifugation at 1000 g for 5 min at 20 ◦C to remove crude proteins. The crude lipids with chloroform phase was collected and transferred into a pre-weighed vial. The extraction procedure was repeated once with addition of 4 mL chloroform and methanol (2:1, v/v) to the aliquot and vortexed for 30 s at room temperature. The chloroform phases from the two extractions were combined and transferred into a pre-weighed vial. Remove the solution from the vial by blowing it with nitrogen gas and remaining lipid extracts were lyophilized overnight (LABCONCO®, Kansas City, USA). Lipids were weighed and total lipids were calculated based on crayfish weight. The composition of fatty acid was determined by an Agilent 7890B+5977A GC/MS (Agilent Technologies Inc., USA) (Yang et al., 2019). Error bars represent the standard deviation (*n* = 3).


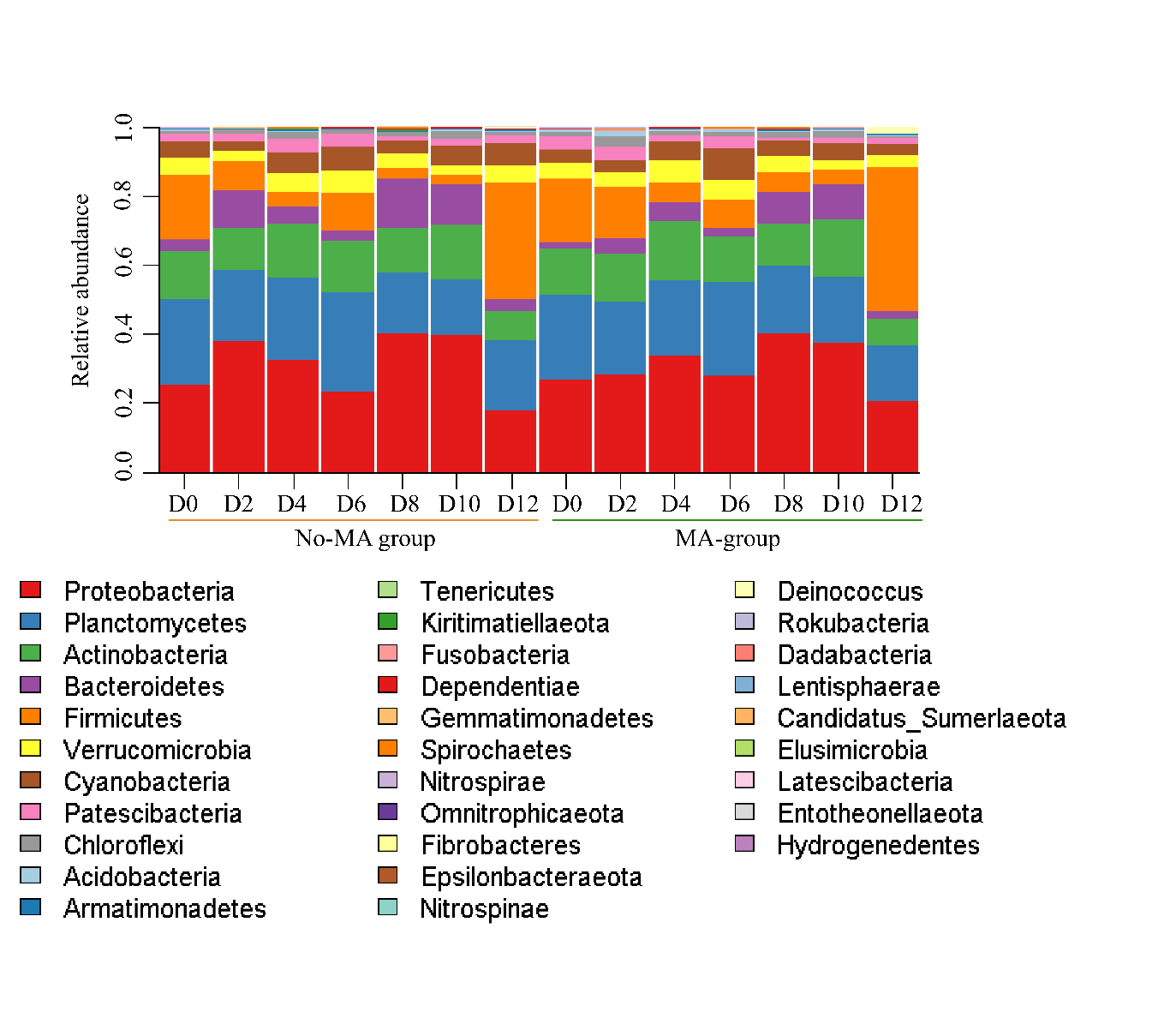


**Figure S1.** Temporal variation in the relative abundance of bacteria at phylum level ranged from the initial day (D0) to day 12 (D12) in the microalgae (MA) and no microalgae (No-MA) application groups.

.

**Figure S2.** Abundance of phytoplankton at phylum level in the microalgae (MA) and no microalgae (No-MA) application groups.

**Figure S3.** Abundance of zooplankton at phylum level in the no microalgae (No-MA) (A) and microalgae (MA) (B) application groups.

**Figure S4.** Fatty acid composition of *P*. *clarkia* in the microalgae (MA) and no microalgae (No-MA) application groups. Error bars represent the standard deviation (n = 3).

**Reference**

Yang, X.; Li, Y.; Li, Y.; Ye, D.; Yuan, L.; Sun, Y.; Han, D.; Hu, Q. Solid Matrix-Supported Supercritical CO_2_ Enhances Extraction of γ-Linolenic Acid from the Cyanobacterium *Arthrospira* (*Spirulina*) *platensis* and Bioactivity Evaluation of the Molecule in Zebrafish. Mar. Drugs 2019, 17(4), 203.
